# Why do users override alerts? Utilizing large language model to summarize comments and optimize clinical decision support

**DOI:** 10.1093/jamia/ocae041

**Published:** 2024-03-07

**Authors:** Siru Liu, Allison B McCoy, Aileen P Wright, Scott D Nelson, Sean S Huang, Hasan B Ahmad, Sabrina E Carro, Jacob Franklin, James Brogan, Adam Wright

**Affiliations:** Department of Biomedical Informatics, Vanderbilt University Medical Center, Nashville, TN 37212, United States; Department of Computer Science, Vanderbilt University, Nashville, TN 37212, United States; Department of Biomedical Informatics, Vanderbilt University Medical Center, Nashville, TN 37212, United States; Department of Biomedical Informatics, Vanderbilt University Medical Center, Nashville, TN 37212, United States; Department of Medicine, Vanderbilt University Medical Center, Nashville, TN 37212, United States; Department of Biomedical Informatics, Vanderbilt University Medical Center, Nashville, TN 37212, United States; Department of Biomedical Informatics, Vanderbilt University Medical Center, Nashville, TN 37212, United States; Department of Medicine, Vanderbilt University Medical Center, Nashville, TN 37212, United States; Department of Biomedical Informatics and Medical Education, University of Washington, Seattle, WA 98195, United States; Department of Pediatrics, Vanderbilt University Medical Center, Nashville, TN 37212, United States; Department of Medicine, Vanderbilt University Medical Center, Nashville, TN 37212, United States; Department of Medicine, Vanderbilt University Medical Center, Nashville, TN 37212, United States; Department of Biomedical Informatics, Vanderbilt University Medical Center, Nashville, TN 37212, United States; Department of Medicine, Vanderbilt University Medical Center, Nashville, TN 37212, United States

**Keywords:** clinical decision support, alert fatigue, health personnel, large language model

## Abstract

**Objectives:**

To evaluate the capability of using generative artificial intelligence (AI) in summarizing alert comments and to determine if the AI-generated summary could be used to improve clinical decision support (CDS) alerts.

**Materials and Methods:**

We extracted user comments to alerts generated from September 1, 2022 to September 1, 2023 at Vanderbilt University Medical Center. For a subset of 8 alerts, comment summaries were generated independently by 2 physicians and then separately by GPT-4. We surveyed 5 CDS experts to rate the human-generated and AI-generated summaries on a scale from 1 (strongly disagree) to 5 (strongly agree) for the 4 metrics: clarity, completeness, accuracy, and usefulness.

**Results:**

Five CDS experts participated in the survey. A total of 16 human-generated summaries and 8 AI-generated summaries were assessed. Among the top 8 rated summaries, five were generated by GPT-4. AI-generated summaries demonstrated high levels of clarity, accuracy, and usefulness, similar to the human-generated summaries. Moreover, AI-generated summaries exhibited significantly higher completeness and usefulness compared to the human-generated summaries (AI: 3.4 ± 1.2, human: 2.7 ± 1.2, *P* = .001).

**Conclusion:**

End-user comments provide clinicians’ immediate feedback to CDS alerts and can serve as a direct and valuable data resource for improving CDS delivery. Traditionally, these comments may not be considered in the CDS review process due to their unstructured nature, large volume, and the presence of redundant or irrelevant content. Our study demonstrates that GPT-4 is capable of distilling these comments into summaries characterized by high clarity, accuracy, and completeness. AI-generated summaries are equivalent and potentially better than human-generated summaries. These AI-generated summaries could provide CDS experts with a novel means of reviewing user comments to rapidly optimize CDS alerts both online and offline.

## Introduction

With the widespread adoption of the electronic health record (EHR), the use of clinical decision support (CDS) systems has also expanded.^1^ CDS systems include a variety of tools such as alerts, reminders, order sets, and documentation templates that provide important insights and recommendations to healthcare providers as they care for patients.^2^ CDS alerts can be categorized as actionable or nonactionable based on whether they provide clear, actionable next steps. For example, an alert about influenza vaccine eligibility that includes a button to order the vaccine is an actionable alert. On the other hand, an alert that simply displays an elevated pediatric early warning score is a nonactionable alert. Well-designed and successfully implemented CDS tools have been reported to play a key role in improving clinical practice and addressing racial and ethnic disparities in healthcare.^3–5^ The foundation of CDS tool design and implementation is the “Five Rights” principle, which includes “the right information, to the right person, in the right intervention format, through the right channel, at the right time in workflow.”^6^ However, a prevalent challenge persists in the real world: approximately 90% of CDS alerts are not accepted, or acted upon, with some of them supported by valid reasons such as low relevance and inappropriate timing.^7^^,^^8^ These common problems occur when CDS is not working as intended and lead to “alert fatigue,” which can seriously jeopardize patient safety.^9^

**Figure 1.**
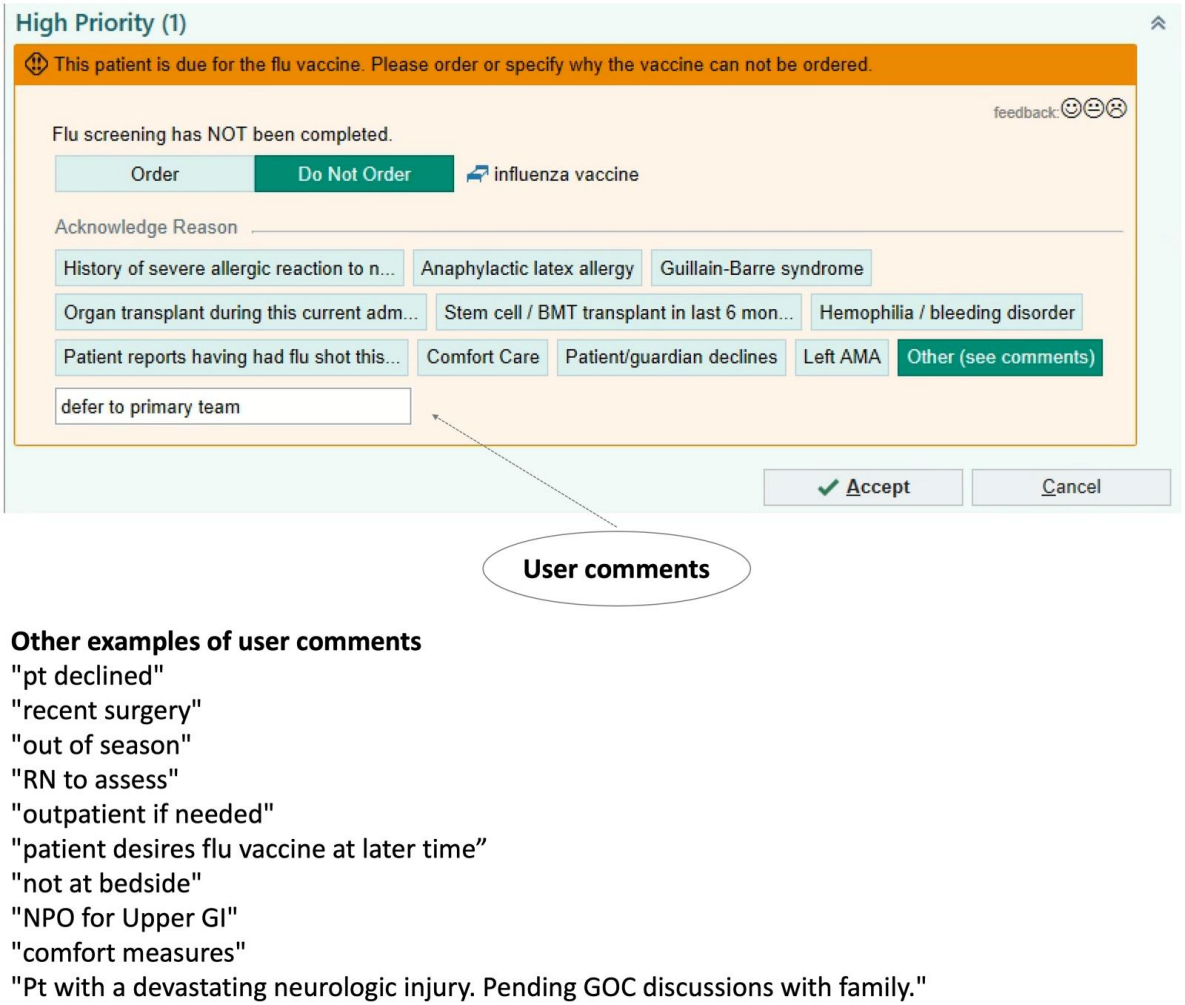
An example of a CDS alert and relevant user comments. CDS = clinical decision support.

Efforts to address this challenge include 2 main categories: (1) the use of manual review and (2) data science-related tools to optimize alerts. For example, Vanderbilt University Medical Center (VUMC) created the “Clickbusters” program, which reduced unnecessary alert triggers by 15% through a detailed 10-step review in collaboration with relevant healthcare providers and CDS experts.^10^ While manual review ensures each insight to be clinically appropriate, it is labor-intensive and may fall short in offering immediate or comprehensive feedback. Conversely, automated tools supported by data science could be used as supplementary support in the manual review process to provide a more sustainable and scalable solution for maintaining the effectiveness of CDS.^11^^,^^12^ Notably, current methods to enhance CDS do not typically take into account the comments or other information provided by healthcare providers when alerts are overridden. This information is commonly unorganized free-text and may contain irrelevant information beyond the override reasons. An example of 1 alert and the corresponding user comments are shown in Figure 1. This alert was designed to notify clinicians to order an influenza vaccine. The user chose to override this alert and left a comment “defer to primary team.” Other examples of user comments for this alert included “patient declined” and “out of season.” Earlier researchers have identified that some free-text comments could be used to determine the reason behind why certain alerts do not work as designed. For example, an alert that suggests an emergency room patient to take insulin is often overridden with a comment such as “patient is already on insulin,” which upon further investigation, researchers found to be a logical error that caused the alert to miss certain previous insulin administrations.^13^ In another study, researchers developed a Naïve Bayes model to classify important override comments and used them to identify malfunctions in 26% of rules in CDS alerts.^14^ However, classification model development requires manual labeling followed by manual review of a sizeable number of comments which are classified into categories. The classification performance of the Naïve Bayes model (Area under the ROC Curve [AUC] = 0.738) also might lead to important comments being incorrectly categorized. In addition to the 2 main categories above, other efforts to improve CDS include investigating the completeness of the data used in the alert logic, performing systematic maintenance of the alert logic, and clarifying the clinical significance of the alert to determine the threshold for interrupting the clinical workflow.^15^

A significant number of free-text comments generated by healthcare providers remain archived in databases, awaiting efficient summarization for timely integration into CDS optimization systems. To illustrate, 85 886 comments were generated at VUMC within a year, with an average of 239 comments per alert. An intuitive solution would be to automatically summarize comments for each alert, thus enabling CDS experts to use these summaries to improve alerts. Advanced generative artificial intelligence (AI) tools such as GPT-4, the instruction-tuned large language models, have shown excellent performance in summarization tasks.^16^ A recent study demonstrated that GPT-4 strongly aligns with humans in the summarization tasks.^17^ One study focused on news summarization and found that the quality of GPT-3 generated summarization was on par with human-written summarizations.^18^ These generative AI tools have also presented great possibilities in clinical tasks, such as summarizing radiology reports and leading expert panel discussion in pediatric palliative care.^19–21^

The objectives of this study were: (1) to evaluate the capability of using generative AI in summarizing alert comments and (2) to determine if the AI-generated summary could be used to improve CDS alerts.

## Methods

### Setting and data collection

We conducted this study at VUMC. This research was reviewed by the Vanderbilt University Institutional Review Board and found to be exempt. We extracted user responses and comments for alerts (BestPractice Advisories [BPAs]) generated from September 1, 2022 to September 1, 2023 from clarity, an epic (Epic Systems, Verona, WI) data warehouse. We grouped alerts into actionable alerts and nonactionable alerts based on whether they provided clear actionable next steps (eg, providing a clickable button to order an influenza vaccine). Notably, if alerts only had links without specific next steps, we grouped them as nonactionable alerts. Within each group, we chose alerts with large numbers of comments, overrides, or a high override rate. We excluded any alerts that had been retired or had fewer than 20 comments. The comment preprocessing involved the following steps: (1) removal of comments with only a single character, (2) exclusion of empty comments, and (3) filtering out comments with the terms “na,” “n/a,” “ok,” or indications of passwords. For alerts with over 500 comments, we made a random selection of 500. If the number of comments was below this threshold, all were retained. In total, we selected 8 alerts (3919 comments) for analysis (Table 1).

**Table 1.** Selected alerts, descriptions and offered actions.

Alert title	Description	Offered action
Travel advisory—ambulatory	To notify clinicians that the initial screening identifies the patient as being at risk for travel exposure to an infectious disease.	Nonactionable
Missing pain score with medication administration	To notify clinicians to document a pain assessment prior to administration.	Nonactionable
Elevated PEWS score	To notify clinicians “patient has an elevated total PEWS score.”	Nonactionable
Signing heparin infusion order modification without PTT order in place	To notify clinicians “per heparin infusion protocol—a repeat PTT is due 6 h after every heparin infusion rate change. After 2 consecutive therapeutic PTTs, repeat PTT every 24 h while in target range. Please select Order and Accept to add a PTT order to your Heparin Modification.	Order PTT
Influenza immunization	To notify clinicians “This patient is due for the flu vaccine. Please order or specify why the vaccine cannot be ordered.”	Order influenza vaccine
Home medications requiring decision	Admission medication reconciliation is incomplete, there are home medications that need a decision.	Review home meds; take action on home meds
VTE prophylaxis	Patient may require VTE prophylaxis—open the panel below for VTE prophylaxis options or select an exclusion reason.	Order VTE prophylaxis panel; order permanent contraindication to VTE prophylaxis for rest of encounter
Naloxone co-prescribing	This patient is at risk for unintentional opioid overdose and a naloxone prescription is required to be offered by TN law.	Order naloxone (Narcan) 4 mg/actuation nasal spray

Abbreviations: PEWS = pediatric early warning score, PTT = partial thromboplastin time, VTE = venous thromboembolism, TN = Tennessee.

### Human-generated summaries

Two physicians (1 internal medicine physician and 1 psychiatrist) reviewed a total of 3919 comments for 8 alerts independently to generate summaries. Both physicians also had a strong background in CDS tools. Prior to generating the summaries, we had a meeting with them to introduce the research project. They knew that their summaries would be used to compare with those generated by AI. For each selected alert, they reviewed the alert logic, the description of alerts, and the screenshots to understand the alert. Then, they reviewed relevant comments and generated their own summaries for these comments.

### AI-generated summaries

For the AI-generated summaries, we used Microsoft Azure’s hosted version of OpenAI GPT-4 large language model. The model was deployed in a protected environment at VUMC, which is approved for usage with protected health information. We used the prompt: “Your task is to analyze comments from clinicians who received a CDS alert but chose not to accept it. The comments are enclosed in double quotes and separated by a line break. Your objective is to categorize and comprehensively summarize the key reasons for not accepting the alert, based on the comments provided by the clinicians. For each summarized reason, list 3-5 representative comments from the document. Please number the summarized reasons and list the comments under each reason.\n =====Comments: [COMMENT].” Each comment was separated by a new line character and used double quotes. The prompt was designed through an iterative process. To improve the effectiveness of prompt, we described the task and the objective to provide contextual information and clarified the format of the inputs and outputs.^22^ Additionally, we asked for several examples under each generated reasons in the summary to ensure that the prompt captured a wide range of comments, thus avoiding premature conclusions based on a limited dataset.

### Expert review of summarizations

We performed a questionnaire survey to rate the human-generated summarizations and AI-generated summarizations. Five CDS experts (4 physicians and 1 pharmacist) participated in the survey. The experts evaluated the quality of summaries written for override comments of CDS alerts following these steps:

1. Carefully read the alert information and relevant comments.
2. Read the proposed summaries A-C (2 human-generated and 1 AI-generated; presented in random order without indication of the source [ie, blinded]).
3. Rate each summary on a scale from 1 (strongly disagree) to 5 (strongly agree) for the 4 metrics:
  - **Clarity**: The summary is logical and easy to understand.
  - **Completeness**: There are no important topics in the comments that the summary misses.
  - **Accuracy**: There are no points in the summary that are not actually found in the comments.
  - **Usefulness**: The summary would be helpful to me as I tried to improve the BPA.
4. Add a comment regarding any hallucinations found in the summary.

### Evaluation

For each metric, we reported the mean and standard deviation. We calculated the overall score by averaging the score across the 4 metrics. We conducted a Mann-Whitney-Wilcoxon test, which is nonparametric, to evaluate the difference between the ratings of AI-generated and human-generated summaries. We considered *P < .*01 as the threshold for statistical significance. Moreover, we measured the intraclass correlation coefficient (ICC) to assess the consistency among raters. An ICC below 0.5 indicates low reliability, between 0.5 and 0.74 indicates moderate reliability, from 0.75 to 0.9 suggests good reliability, and above 0.9 signifies excellent reliability.^23^ We used Python 3.8 for statistical analysis. Descriptive statistics were also reported to characterize the survey participants, including their clinical specialties, roles, and years of CDS experience.

## Results

This survey involved 5 CDS experts with backgrounds in internal medicine, pharmacy, geriatric medicine, and pediatrics. On average, they had 6 years of clinical experience with the EHR (since graduation from medical school). They were currently in practice and received CDS alerts on a daily basis. Details about the survey participants can be found in Table S1. The survey demonstrated good reliability, as indicated by an ICC value of 0.75.

### Examples of AI-generated summaries and human-generated summaries

Among the top 8 rated summaries (based on the overall score), five were generated by GPT-4 and three were generated by humans. Table 2 lists these generated summaries and their ratings for clarity, completeness, accuracy, and usefulness. The remaining generated summaries were listed in Table S2.

**Table 2.** Top 8 summaries and their ratings for clarity (C1), completeness (C2), accuracy (A), usefulness (U), and overall (O).

	Alert	Summary	C1	C2	A	U	O
AI	VTE prophylaxis	Patient is undergoing or is scheduled for a procedure Bleeding or coagulation issues In palliative or hospice care Patient is refusing treatment Ambulatory or physically active Not the primary caregiver or is unfamiliar with the patient Low platelet count Planned discharge or is being discharged	5 (0)	4.2 (0.5)	4.6 (0.6)	4.6 (0.6)	4.6 (0.3)
AI	Missing pain score with medication administration	Pain management Fever or temperature concerns Certain procedures or treatments required the clinicians to dismiss the alert Patient or family preferences Patient’s physiological cues or behavioral signs Premedication needs Clinicians also mentioned various assessment scores or referred to patient charting as reasons for not accepting the alert System or documentation issues Patient's inability to self-report	4.2 (1.3)	4.2 (0.5)	4.6 (0.9)	4.2 (0.8)	4.3 (0.7)
AI	Naloxone Co-prescribing	Patient already has Naloxone/Narcan prescription Patient transferred to another facility Prescription not necessary/not indicated Patient declined the offer Prescription will be addressed later Patient is no longer taking the medication Prescription already ordered/sent Patient under the care of another provider	4.8 (0.5)	3 (1.4)	4.8 (0.5)	4.6 (0.6)	4.3 (0.5)
AI	Elevated PEWS score	Notification of medical team or charge nurse Patient's current condition is stable or at baseline Patient's emotional state affecting vitals Medical team already at bedside or aware Need for reassessment or monitoring	4.2 (0.8)	3.6 (1.1)	4.6 (0.6)	4.4 (0.9)	4.2 (0.7)
AI	Signing heparin infusion order modification without PTT order in place	Already ordered or scheduled Following protocol Monitoring other parameters, such as Anti-Xa and heparin levels instead of PTT Timing or scheduling issues Therapeutic levels Manual ordering or adjustments Error or duplication Stopping or changing treatment Miscellaneous reasons such as patient transfer, procedure, or comfort care	4.2 (1.3)	3.8 (1.1)	4.6 (0.6)	4.2 (0.8)	4.2 (0.7)
Human	Travel advisory—ambulatory	Patient wearing mask Prior positive test out of isolation window COVID test to be completed today Negative home COVID test No respiratory symptoms	4.8 (0.5)	3.4 (1.1)	4.4 (1.3)	4.2 (0.5)	4.2 (0.7)
Human	Flu immunization	Patient declined for various reasons, deferred until follow-up, or had been discharged Patient was a surgical patient/post-op/peri-op Patient had recent illness or acute medical process including being flu or Covid+ Provider was not primary team and/or deferred to PCP Flu vaccine was being considered, pending, and/or to be discussed with patient Due to a medical process/diagnoses, flu vaccine deferred to specialists Another logistical reasons (late-season, vaccine not available, or given previously)	4.2 (1.3)	3.4 (1.3)	4.8 (0.5)	4.2 (0.8)	4.2 (0.7)
Human	VTE prophylaxis	Active bleeding Comfort measures Patient refuses Thrombocytopenia Planned procedure Coagulopathy	5 (0)	2.6 (1.1)	4.6 (0.6)	4.2 (0.8)	4.1 (0.3)

Abbreviations: VTE = venous thromboembolism, PEWS = Pediatric early warning score, PTT = partial thromboplastin time.

Values are given as mean (SD).

### Results of expert review of AI-generated suggestions and human-generated suggestions

**Figure 2.**
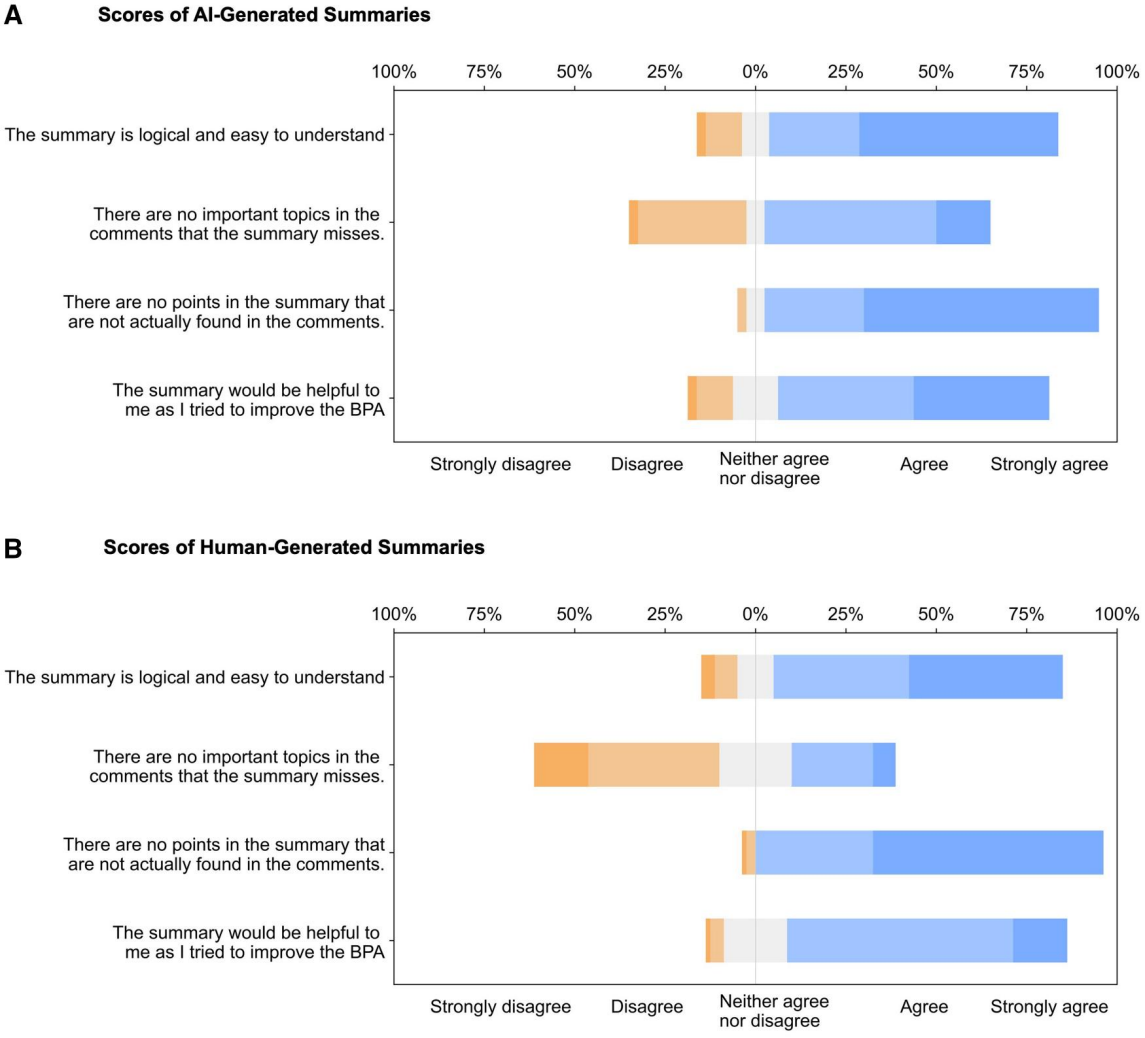
Stacked bar charts of the scores for clarity, completeness, accuracy, and usefulness of AI-generated summaries (A) and human-generated summaries (B). AI = artificial intelligence.

Participants evaluated 8 AI-generated and 16 human-generated summaries corresponding to 8 alerts. Every AI-generated summary scored at least 3 in the overall metric, with the highest being 4.6 ± 0.3. The AI-generated summaries typically received agreement for clarity and usefulness, while completeness received a neutral response, neither agree nor disagree. Accuracy, however, tended toward strong agreement. Figure 2A displays a stacked bar chart of the AI-generated summaries' scores across each metric, while Figure 2B presents the same for human-generated summaries. Most of the AI-generated summaries, 87.5% (7 out of 8) and 75% (12 out of 16) of human-generated summaries scored 4 or above for clarity. For completeness, 75% of AI-generated and 37.5% of human-generated summaries scored 3 or higher. All summaries from both AI and humans achieved a score of 4 or above for accuracy. This study did not detect any instances of AI hallucination. Regarding usefulness, 87.5% of AI-generated and 93.75% of human-generated suggestions were rated 3 or higher.

AI-generated summaries were rated significantly higher than human-generated summaries in terms of completeness (AI: 3.4 ± 1.2, human: 2.7 ± 1.2, *P* = .001). Other metrics were rated similarly. AI-generated summaries achieved a score of 4.2 ± 1.1 for clarity, 4.5 ± 0.7 for accuracy, 4 ± 1.1 for usefulness, and an overall score of 4 ± 0.8. Meanwhile, human-generated summaries were rated with a clarity score of 4.1 ± 1.1, accuracy of 4.5 ± 0.7, usefulness of 3.9 ± 0.8, and an overall score of 3.8 ± 0.6. No significant differences were found in the human-generated summaries (Table 3).

**Table 3.** Means and SD for survey questions rated on a 5-point Likert scale, with 1 indicating “strongly disagree” and 5 indicating “strongly agree.”

	AI-generated summaries mean (SD)	Human-generated summaries mean (SD)	*P*
**Clarity**: The summary is logical and easy to understand.	4.2 (1.1)	4.1 (1.1)	.176
**Completeness**: There are no important topics in the comments that the summary misses.	3.4 (1.2)	2.7 (1.2)	.001
**Accuracy**: There are no points in the summary that are not actually found in the comments.	4.5 (0.7)	4.5 (0.7)	.499
**Usefulness**: The summary would be helpful to me as I tried to improve the alert.	4.0 (1.1)	3.9 (0.8)	.011

## Discussion

In this study, we explored the feasibility of using GPT-4 to summarize user override comments on CDS alerts, comparing its performance with human-generated summaries. AI-generated summaries demonstrated high levels of clarity, accuracy, and usefulness, similar to the human-generated summaries. Moreover, AI-generated summaries exhibited significantly higher completeness and usefulness compared to the human-generated summaries.

The quality of human-generated summaries might be affected by several factors. One example is the anchoring effect, where only certain words are considered in generating summaries. For instance, in the case of the VTE prophylaxis alert, several comments like “patient is ambulatory” and “patient up and walking” were adeptly identified and classified by GPT-4 as “ambulatory or physically active.” On the other hand, 1 human-generated summary did not include this point, while another confused “ambulatory” as referring to a patient location “patient location (ambulatory).” Ambulatory in the comments was referring to patients being ambulatory (ie, walking around) and not needing VTE prophylaxis, not to their being in an outpatient setting (the alert only fired in the inpatient setting). Reviewing a long list of comments is tedious, and humans may be more likely to accidentally omit a category due to the sheer number of comments listed.

Creating fewer, broad, vague categories might be logical/correct but less helpful when trying to improve the BPA than having more, detailed categories in the summary. For example, a part of an AI-generated summary “specific medical circumstances” may be accurate yet too ambiguous to be actionable. In contrast, a human-generated summary like “the time check of PTT recommended by the BPA was not accurate” pinpoints a specific issue needing improvements. The prompt could be engineered to deliver more specific, actionable categories in the AI-generated summary. Therefore, future studies could consider the use of generative AI technology to assist in manual review—by providing a summary and flagging comments that may require human review.

The original AI-generated summaries included example comments under each category, which we removed in the survey to align with the format of the human-generated summaries and ensure a blind evaluation. A participant suggested that “it would be more helpful if the model gave the category and then some examples.”

**Figure 3.**
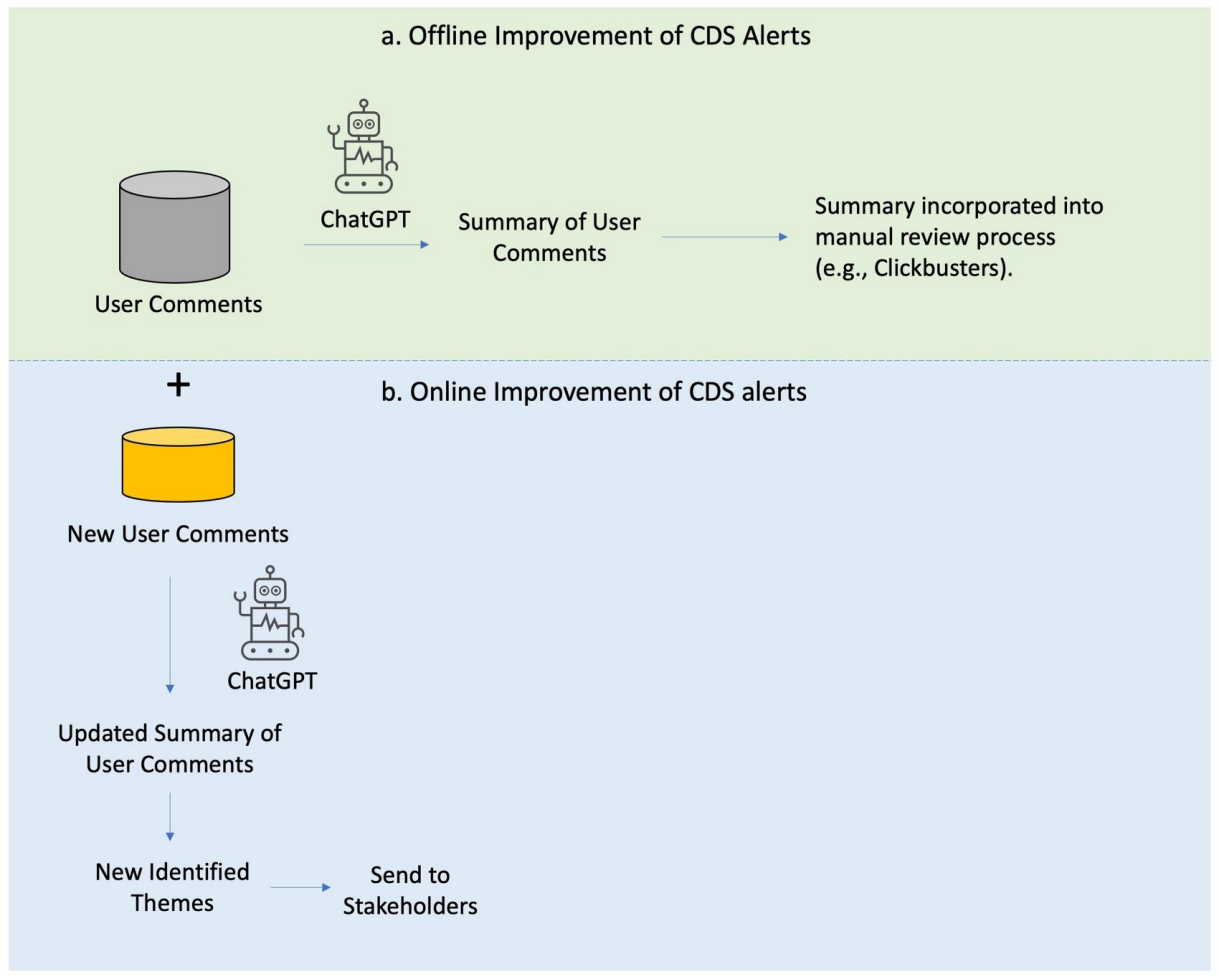
Using AI-generated summary of user comments to improve CDS alerts offline and online. AI = artificial intelligence, CDS = clinical decision support.

AI-generated summaries could help CDS experts to improve alerts both offline and online (Figure 3). GPT-4 allows for the summarization of millions of user comments, offering the possibility to integrate user comments into a CDS alert review process, such as Clickbusters. In addition, summarizing user comments using GPT-4 provides a way to automatically monitor user comments and identify new themes to send to stakeholders. It should be emphasized that the integration of AI-generated summaries of user reviews is a valuable component of CDS alert optimization, not a replacement for the entire optimization process. While user feedback is an important source of immediate insight, it may not capture all of the factors that influence user acceptance of alerts.

In addition, the process of using user comments to refine CDS alerts could serve as an incentive for increased user engagement. The expectation that their comments will be promptly and effectively used to refine CDS alerts may encourage more substantive comments. This potential for increased engagement may extend the applicability of our findings to a broader range of institutions, fostering a more interactive and responsive environment between CDS experts and healthcare providers. Future studies include: (1) a comprehensive analysis of using AI-generated summaries of all user comments to improve CDS alerts, and (2) a comparative analysis of the potential impact on user behavior and patient outcomes.

When discussing how GPT-4 can be employed to summarize CDS alerts, the logistics of its implementation need to be taken into consideration. At VUMC, between September 2022 and September 2023, we identified 195 alerts with a minimum of 20 comments, averaging around 434 comments. Given an average comment token size of 6.02 tokens and an average response length of 487 tokens in our evaluation dataset, using GPT-4 Turbo as of November 21, 2023, which has a token limit of 128k, would require generating 196 prompts with approximately 530 722 input tokens and around 95 452 output tokens to process all user comments. At the current rate of $0.01 per 1000 tokens for input and $0.03 per 1000 tokens for output, the total cost would amount to $8.17, which is quite inexpensive when compared with the possibility of asking expert clinicians to do the same review. Using GPT-3.5 Turbo, the total cost would be even lower at $0.73.

### Limitations

There are several limitations to our study. First, we evaluated the generated summaries from CDS experts’ perspectives. The impact of generated summaries from user comments on the actual change of CDS alerts or relevant patient outcomes was not evaluated. Second, as a preliminary study, we randomly selected 500 comments for each alert. If we had used all comments, the AI-generated summaries and the human-generated summaries may have differed. Third, in this study, 2 physicians generated summaries. The quality of human-generated summaries might be improved with the involvement of a larger number of physicians in the summarization process. Fourth, this study was conducted at an institution with a large number of user comments for CDS alert. The generalizability of this study to other institutions remains unknown, especially in smaller medical centers.

## Conclusion

End-user comments provide clinicians’ immediate feedback to CDS alerts and can serve as valuable data resource for optimizing CDS interventions. Traditionally, these comments may not be considered in the CDS review process due to their unstructured nature, large volume, and the presence of redundant or irrelevant content. Our study demonstrates that GPT-4 is capable of distilling these comments into summaries characterized by high clarity, accuracy, and completeness. AI-generated summaries are equivalent and potentially better than human-generated summaries. These AI-generated summaries could provide CDS experts with a novel means of reviewing user comments to rapidly optimize CDS alerts both online and offline.

## Supplementary Material

ocae041_Supplementary_Data

## Data Availability

The user comments used in this study cannot be shared publicly due to patient healthcare data privacy protection requirements. The AI-generated summaries, human-generated summaries, and the relevant alert content were reported in the Supplementary Material.

## References

[1] Parasrampuria S , Henry J. Hospitals’ use of electronic health records data, 2015-2017. ONC Data Br. 2019;46:1-13. 39680702

[2] Wright A , Sittig DF, Ash JS, et al Development and evaluation of a comprehensive clinical decision support taxonomy: comparison of front-end tools in commercial and internally developed electronic health record systems. J Am Med Informatics Assoc. 2011;18(3):232-242. 10.1136/amiajnl-2011-000113 PMC3078666 21415065

[3] Thomas Craig KJ , Fusco N, Lindsley K, et al Rapid review: identification of digital health interventions in atherosclerotic-related cardiovascular disease populations to address racial, ethnic, and socioeconomic health disparities. Cardiovasc Digit Health J. 2020;1(3):139-148. 10.1016/j.cvdhj.2020.11.001 35265886 PMC8890337

[4] Wright A , Phansalkar S, Bloomrosen M, et al Best practices in clinical decision support: the case of preventive care reminders. Appl Clin Inform. 2010;1(3):331-345. 10.4338/ACI-2010-05-RA-0031 21991299 PMC3189503

[5] Douthit BJ , McCoy AB, Nelson SD. The impact of clinical decision support on health disparities and the digital divide. Yearb Med Inform. 2023;32(1):169-178. Published Online First: 6 July 10.1055/s-0043-1768722 37414030 PMC10751127

[6] Osheroff J. Improving Outcomes with Clinical Decision Support: An Implementer’s Guide. 2nd ed. Chicago: HIMSS Publishing; 2012. 10.4324/9781498757461

[7] Seidling HM , Klein U, Schaier M, et al What, if all alerts were specific—estimating the potential impact on drug interaction alert burden. Int J Med Inform. 2014;83(4):285-291. 10.1016/j.ijmedinf.2013.12.006 24484781

[8] van der Sijs H , Aarts J, Vulto A, et al Overriding of drug safety alerts in computerized physician order entry. J Am Med Informatics Assoc. 2006;13(2):138-147. 10.1197/jamia.M1809 PMC1447540 16357358

[9] Wright A , Aaron S, Seger DL, et al Reduced effectiveness of interruptive drug-drug interaction alerts after conversion to a commercial electronic health record. J Gen Intern Med. 2018;33(11):1868-1876. 10.1007/s11606-018-4415-9 29766382 PMC6206354

[10] McCoy AB , Russo EM, Johnson KB, et al Clinician collaboration to improve clinical decision support: the Clickbusters initiative. J Am Med Informatics Assoc. 2022;29(6):1050-1059. 10.1093/jamia/ocac027 PMC9093034 35244165

[11] Liu S , Kawamoto K, Del Fiol G, et al The potential for leveraging machine learning to filter medication alerts. J Am Med Informatics Assoc. 2022;29(5):891-899. 10.1093/jamia/ocab292 PMC9006688 34990507

[12] Liu S , Wright AP, Patterson BL, et al Using AI-generated suggestions from ChatGPT to optimize clinical decision support. J Am Med Informatics Assoc. 2023;30(7):1237-1245. 10.1093/jamia/ocad072 PMC10280357 37087108

[13] Wright A , Ai A, Ash J, et al Clinical decision support alert malfunctions: analysis and empirically derived taxonomy. J Am Med Inform Assoc. 2018;25(5):496-506. 10.1093/jamia/ocx106 29045651 PMC6019061

[14] Aaron S , McEvoy DS, Ray S, Hickman T, Wright A. Cranky comments: detecting clinical decision support malfunctions through free-text override reasons. J Am Med Inform Assoc. 2019;26(1):37-43. 10.1093/jamia/ocy139 30590557 PMC6308015

[15] Phansalkar S , van der Sijs H, Tucker AD, et al Drug—Drug interactions that should be non-interruptive in order to reduce alert fatigue in electronic health records. J Am Med Informatics Assoc. 2013;20(3):489-493. 10.1136/amiajnl-2012-001089 PMC3628052 23011124

[16] Zhang H , Liu X, Zhang J. SummIt: iterative text summarization via ChatGPT. Accessed October 11, 2023. https://chat.openai.com/chat

[17] Liu Y , Iter D, Xu Y, et al G-Eval: NLG evaluation using GPT-4 with better human alignment [Published Online First: March 29, 2023]. Accessed October 11, 2023. https://arxiv.org/abs/2303.16634v3

[18] Zhang T , Ladhak F, Durmus E, et al Benchmarking large language models for news summarization [Published Online First: January 31, 2023]. Accessed October 11, 2023. https://arxiv.org/abs/2301.13848v1

[19] Ma C , Wu Z, Wang J, et al ImpressionGPT: an iterative optimizing framework for radiology report summarization with ChatGPT [Published Online First: April17, 2023]. Accessed October 11, 2023. https://arxiv.org/abs/2304.08448v2

[20] Almazyad M , Aljofan F, Abouammoh NA, et al Enhancing expert panel discussions in pediatric palliative care: innovative scenario development and summarization with ChatGPT-4. Cureus. 2023;15(4):e38249. 10.7759/cureus.38249 37122982 PMC10143975

[21] Liu J , Wang C, Liu S. Utility of ChatGPT in clinical practice. J Med Internet Res. 2023;25:e48568. 10.2196/48568 37379067 PMC10365580

[22] Meskó B. Prompt Engineering as an Important Emerging Skill for Medical Professionals: Tutorial. J Med Internet Res. 2023;25:e50638. 10.2196/50638 37792434 PMC10585440

[23] Koo TK , Li MY. A guideline of selecting and reporting intraclass correlation coefficients for reliability research. J Chiropr Med. 2016;15(2):155-163. 10.1016/j.jcm.2016.02.012 27330520 PMC4913118

