# Supplementary material for: Why do users override alerts? Utilizing large language model to summarize comments and optimize clinical decision support
Source: J Am Med Inform Assoc. 2024 Mar 7;31(6):1388–96. doi: 10.1093/jamia/ocae041 (PMC11105133; doi:10.1093/jamia/ocae041)
Supplement: ocae041_Supplementary_Data [file ocae041_supplementary_data.docx]

**Alert Information:**

Travel Advisory – Ambulatory
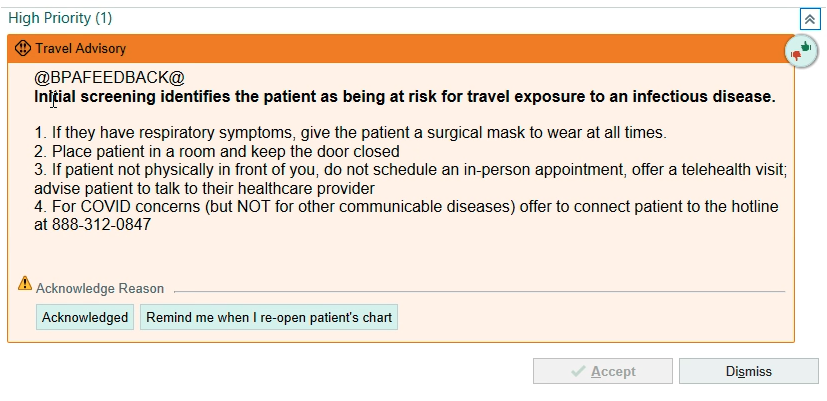


Missing Pain Score with Medication Administration


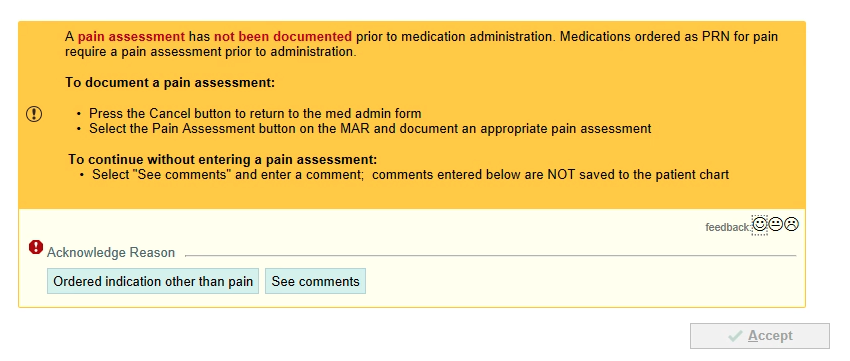


Elevated PEWS Score
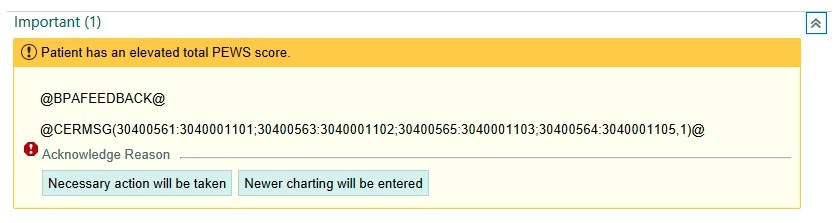


To alert that patient has an elevated total PEWS score. This alert is shown to Registered Nurse and Licensed Nurse.

Signing Heparin Infusion Order Modification without PTT Order in Place
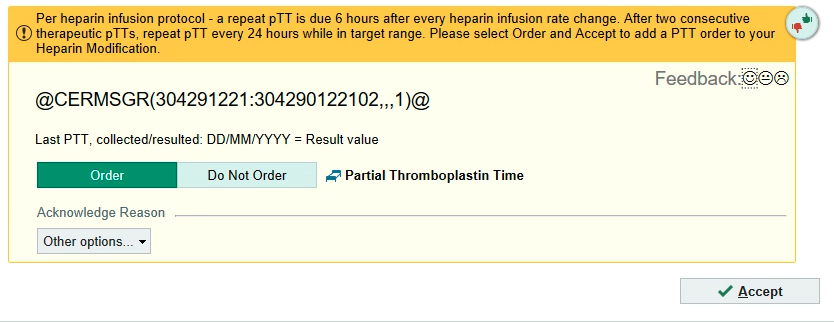


This BPA is used to help remind Nurses when they use the modify order link to adjust the Heparin infusion on the patient, that Protocol requires them to place an order for a PTT order 6 hours after the modification of the Heparin infusion.  The BPA triggers with the sign order trigger and suggests the user accept the new order for PTT as well, that order is built with the defaults in the BPA LPF, with defaults already set for 6 hours.  The Smart Link is attached to an ETX Wrapper that has a rule in it that checks if there are other active PTT orders on the patient, but are out of range, and adds a message in the display that says "This patient has an order for PTT at ##:## Time, consider canceling that PTT order and accept this one."  It also has the BPA Feedback Faces and RedCap link.

Flu Immunization


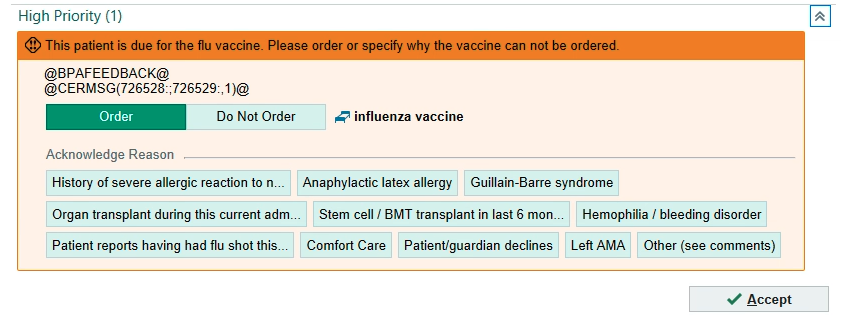


This patient is due for the flu vaccine. Please order or specify why the vaccine cannot be ordered.

Home Medications Requiring Decision


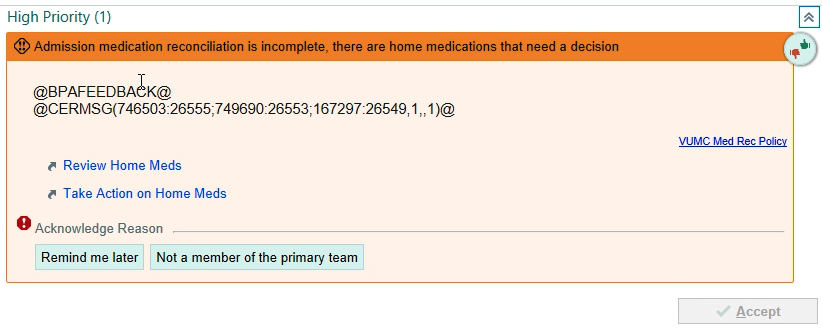


Admission medication reconciliation is incomplete, there are home medications that need a decision.

VTE Prophylaxis

Patient may require VTE prophylaxis - open the panel below for VTE prophylaxis options or select an exclusion reason.


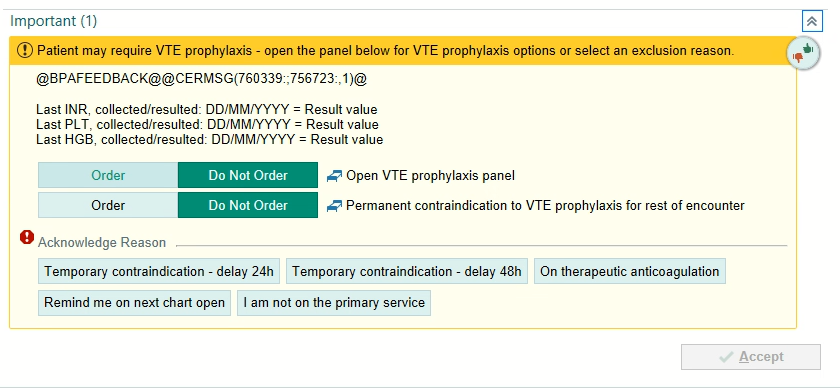


Naloxone Co-prescribing

This patient is at risk for unintentional opioid overdose due to the following and a naloxone prescription is required to be offered by TN law.
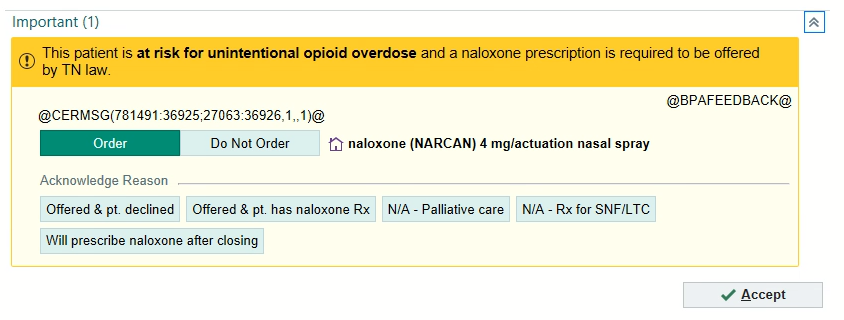


Appendix Table 1**.** Characteristic of 5 CDS experts participating in the survey.

| **Gender** |  |
| --- | --- |
| Male | 2 |
| Female | 3 |
| **Clinical Specialty** |  |
| Internal medicine (primary care) | 1 |
| Internal medicine (hospital medicine) | 1 |
| Internal medicine (geriatrics) | 1 |
| Pharmacy | 1 |
| Pediatrics | 1 |
| **Average Years of Clinical Experience** | 6 |

| Appendix Table 2. AI-generated summaries and human-generated summaries. | | | | | | | | | | | | | |
| --- | --- | --- | --- | --- | --- | --- | --- | --- | --- | --- | --- | --- | --- |
| Alert | | Input token | | Output token | | AI-generated summary | Human-generated summary 1 | | | Human-generated summary 2 | | | |
| Travel Advisory – Ambulatory | | 2956 | | 424 | | • COVID-19 Status • Testing and Quarantine Measures • Use of Personal Protective Equipment (PPE) • Doctor’s Awareness and Involvement • Patients' Misunderstanding or Mistakes in Providing Information • Patient Isolation and Precautions | • Patient wearing mask • Prior positive test out of isolation window • COVID test to be completed today • Negative home COVID test • No respiratory symptoms | | | • Precautions or action had been taken • Patient had already taken action  • Advisory not relevant (patient outside of symptom window, prior hospitalization or positive test) • Error occurred (patient not actually COVID positive, no recent travel) | | | |
| Missing Pain Score with Medication Administration | | 2939 | | 701 | | • Pain management • Fever or temperature concerns • Certain procedures or treatments required the clinicians to dismiss the alert. • Patient or family preferences • Patient's physiological cues or behavioral signs • Pre-medication needs • Clinicians also mentioned various assessment scores or referred to patient charting as reasons for not accepting the alert. • System or documentation issues • Patient's inability to self-report | | | • Pain score reported in the comment itself  • Patient febrile • Premedication for dressing change or wound care • Documented in flowsheets • RASS, CPOT scores | | | • Fever/Temperature • stating that pain score was documented already • ordered per provider recommendation, for some other medical reason such as tachycardia/tachypnea/anxiety/agitation,  • related to an upcoming procedure/imaging | |
| Elevated PEWS Score | | 3415 | | 622 | | • Notification of medical team or charge nurse • Patient's current condition is stable or at baseline • Patient's emotional state affecting vitals • Medical team already at bedside or aware • Need for reassessment or monitoring | | | • Rapid response called • Team notified • Charge nurse notified • Team aware of clinical status • Team already at bedside • Will continue to monitor | | | • Clinicians were aware of patient's status with plans for increased level of monitoring or continued monitoring • Patient's status as being stable.  • Patient was 1) upset/anxious, 2) at baseline), 3) recent treatment for PEWS score had occurred | |
| Signing Heparin Infusion Order Modification without PTT Order in Place | | 3220 | | 512 | | • Already ordered or scheduled • Following protocol • Monitoring other parameters, such as Anti-Xa and heparin levels instead of PTT  • Timing or scheduling issues • Therapeutic levels • Manual ordering or adjustments • Error or duplication • Stopping or changing treatment • Miscellaneous reasons such as patient transfer, procedure, or comfort care. | | | • PTT already ordered • Will order new PTT • Trending factor Xa  • Did not change rate | | | • Follow anti-Xa levels or pharmacy protocol for monitoring Heparin • A PTT order had already been satisfied in some capacity  • The time check of PTT recommended by the BPA was not accurate.  • Heparin was set to be completed prior to next needed PTT lab, or occasionally that PTT was needed sooner. | |
| Flu Immunization | | 2839 | | 424 | | • Current or Recent Illness/Surgery • Defer to Primary Care Provider (PCP) • Patient Refusal or Decline • Timing/Seasonality • Protocol Requirements • Unable to Consent • Patient will Follow-up Outpatient | | | • Defer to after discharge with PCP • Actively infected • Surgical patient • Patient declines • Hospice or comfort care • Out of season • Immunocompromised | | | • Patient declined for various reasons, deferred until follow-up, or had been discharged • Patient was a surgical patient/post-op/peri-op • Patient had recent illness or acute medical process including being flu or Covid + • Provider was not primary team and/or deferred to PCP • Flu vaccine was being considered, pending, and/or to be discussed with patient • Due to a medical process/diagnoses, flu vaccine deferred to specialists • Another logistical reasons (late-season, vaccine not available, or given previously) | |
| Home Medications Requiring Decision | | 2258 | | 198 | | - Deferred to later or another provider - Currently in surgery/procedure - Inappropriate timing/other priorities - Unrelated or nonsensical comments - Specific medical circumstances | | | • Attending does not want to see this BPA • Consultant  • Comfort care • Anesthesia team • Surgery team • Later • Pending pharmacy consult | | | • Review will be done with orders, not able to complete now, review pending, or review would be done later • Deferred BPA or stated contraindications • Provider not primary team or correct provider for review • Location of patient was not good for review | |
| VTE Prophylaxis | | 3054 | | 526 | | • Patient is undergoing or is scheduled for a procedure • Bleeding or coagulation issues • In palliative or hospice care • Patient is refusing treatment • Ambulatory or physically active • Not the primary caregiver or is unfamiliar with the patient • Low platelet count • Planned discharge or is being discharged | | | • Active bleeding • Comfort measures • Patient refuses • Thrombocytopenia • Planned procedure • Coagulopathy | | | • Medical reasons (active bleeding, potential upcoming operation, or lab values (platelets, hemoglobin))  • Patient declining • Patient location (post-op, ambulatory) • Patient status (palliative/comfort care/deceased)  • Not a part of primary team and/or where a consultant | |
| Naloxone Co-prescribing | | 3795 | | 494 | | - Patient already has Naloxone/Narcan prescription - Patient transferred to another facility - Prescription not necessary/ not indicated - Patient declined the offer - Prescription will be addressed later - Patient is no longer taking the medication - Prescription already ordered/sent - Patient under the care of another provider | | | • Already has home supply from prior prescription • Already ordered • Patient refused • Going to facility | | | • Naloxone already being ordered  • Patient already has naloxone by another means • Opioid was low risk  • Patient previously tolerated current medication regimen • Patient disposition location not consistent with prescribing of naloxone  • Patient declined, patient would be offered, patient no longer took medication leading to concern for Naloxone • Patient had a previous bad reaction/allergy to Naloxone | |

Appendix Table 3. Ratings for clarity, completeness, accuracy, usefulness, and overall**.**

| **BPA title** |  | Clarity | Completeness | Accuracy | Usefulness | Overall | P |
| --- | --- | --- | --- | --- | --- | --- | --- |
| Home Medications Requiring Decision | AI | 4.0±1.2 | 2.8±1.3 | 4.6±0.5 | 3.6±1.1 | 3.8±0.7 | 0.53 |
|  | Human | 3.8±1.3 | 2.6±1.3 | 4.4±1.0 | 3.3±1.3 | 3.5±1.0 |  |
| Flu Immunization | AI | 4.6±0.5 | 2.8±1.1 | 4.6±0.5 | 3.8±1.1 | 4.0±0.6 | 0.55 |
|  | Human | 4.3±1.1 | 3.2±1.4 | 4.7±0.5 | 4.2±0.6 | 4.1±0.6 |  |
| VTE Prophylaxis | AI | 5.0±0.0 | 4.2±0.4 | 4.6±0.5 | 4.6±0.5 | 4.6±0.3 | 0.002 |
|  | Human | 4.4±0.8 | 2.8±1.2 | 4.3±1.3 | 4.1±0.7 | 3.9±0.4 |  |
| Naloxone Co-prescribing | AI | 4.8±0.4 | 3.0±1.4 | 4.8±0.4 | 4.6±0.5 | 4.3±0.5 | 0.01 |
|  | Human | 3.7±1.2 | 2.7±1.3 | 4.7±0.5 | 4.0±0.7 | 3.8±0.5 |  |
| Travel Advisory – Ambulatory | AI | 2.6±1.1 | 3.0±1.4 | 4.0±1.4 | 2.4±1.1 | 3.0±1.0 | 0.02 |
|  | Human | 3.7±1.5 | 2.9±1.1 | 4.5±1.0 | 3.7±0.8 | 3.7±0.8 |  |
| Elevated PEWS Score | AI | 4.2±0.8 | 3.6±1.1 | 4.6±0.5 | 4.4±0.9 | 4.2±0.7 | 0.12 |
|  | Human | 4.2±0.9 | 2.6±0.8 | 4.6±0.5 | 3.9±0.6 | 3.8±0.4 |  |
| Missing Pain Score with Medication Administration | AI | 4.2±1.3 | 4.2±0.4 | 4.6±0.9 | 4.2±0.8 | 4.3±0.6 | 0.04 |
|  | Human | 4.3±0.7 | 2.3±1.2 | 4.6±0.5 | 3.9±0.6 | 3.8±0.4 |  |
| Signing Heparin Infusion Order Modification without PTT Order in Place | AI | 4.2±1.3 | 3.8±1.1 | 4.6±0.5 | 4.2±0.8 | 4.2±0.6 | 0.04 |
|  | Human | 4.3±0.8 | 2.4±1.1 | 4.6±0.5 | 3.8±0.4 | 3.8±0.3 |  |
